# Urethrocavernocutaneous Fistula in a Patient With an Inflatable Penile Prosthesis Following Urethral and External Catheter Trauma: A Case Report

**DOI:** 10.7759/cureus.95143

**Published:** 2025-10-22

**Authors:** John Gibson, Michael George, Luke Foster, Rans Nadir, Anas Hattab, Vaibhav Modgil, Ian Pearce, Theodora Stasinou, Peter Grice

**Affiliations:** 1 Department of Urology, Manchester University NHS Foundation Trust, Manchester, GBR; 2 Department of Urology, Manchester Andrology Research Collaborative, Manchester, GBR; 3 Department of Radiology, Manchester University NHS Foundation Trust, Manchester, GBR; 4 Faculty of Biology, Medicine, and Health, The University of Manchester, Manchester, GBR; 5 Department of Urology, Manchester Royal Infirmary, Manchester, GBR

**Keywords:** catheter-associated trauma, inflatable penile prosthesis, nakaseomyces glabratus, prosthesis-related infections, urethrocavernocutaneous fistula

## Abstract

We report a case of urethrocavernocutaneous fistula in a 70-year-old man with a long-standing inflatable penile prosthesis (IPP), attributed to traumatic urethral catheterisation and external pressure injury from an external (conveen) catheter. His significant co-morbidities, including type 1 diabetes mellitus and peripheral vascular disease, likely contributed to impaired tissue healing and susceptibility to infection. During a medical admission, he developed a penile infection with purulent discharge from the glans and mid-shaft and a draining sinus at the previous infrapubic incision. MRI demonstrated peri-prosthetic fluid collections and a 7 × 4 cm abscess, while intraoperative findings confirmed a urethrocavernocutaneous fistula. The device was explanted, and urinary diversion was established via suprapubic and urethral catheters. Cultures grew *Nakaseomyces glabratus*, adding further complexity to the infection. This case illustrates the serious risks of both urethral and external catheter use in patients with IPPs, particularly those with significant co-morbidities. This case underscores the importance of recognising the presence of an IPP early, carefully weighing the need for catheterisation in such patients, ensuring prompt surgical management of infection, and developing clearer guidance on catheter use to minimise avoidable morbidity and device loss. To our knowledge, this represents the first reported case of a urethrocavernocutaneous fistula complicating an IPP, highlighting its rarity and clinical significance.

## Introduction

Inflatable penile prostheses (IPPs) are a well-established surgical solution for men with erectile dysfunction and have demonstrated high long-term satisfaction and functional outcomes [1]. Nevertheless, complications such as infection, erosion, and mechanical failure remain clinically significant, albeit uncommon [2,3]. In particular, catheter-associated trauma, whether from urethral or external devices, has been identified as a potentially modifiable risk factor for prosthesis-related morbidity [4-6]. Patients with diabetes, vascular disease, or impaired mobility are especially vulnerable [7-9].

Although urethral erosion secondary to Foley catheterisation has been described [4], external catheter devices, such as Conveen catheters, are often assumed to be safer alternatives. Emerging reports, however, challenge this perception, showing that sustained pressure or friction from urethral or external catheters may cause ischaemia and mucosal breakdown, ultimately leading to serious complications including penile necrosis [5], gangrene [9,10], and fistula formation [8]. These observations highlight the potential for catheter-associated trauma to compromise outcomes in patients with penile prostheses.

Here, we present, to our knowledge, the first documented case of a urethrocavernocutaneous fistula complicating an IPP, arising from the combined effects of urethral catheter trauma and external catheter ischaemic injury.

## Case presentation

A 70-year-old male with a background of type 1 diabetes mellitus, peripheral vascular disease (with previous digital amputations and delayed wound healing), cerebrovascular accident, upper gastrointestinal bleeding, diabetic retinopathy, and ischaemic cardiomyopathy presented with signs of penile infection during a medical admission. He had undergone an IPP insertion over 10 years previously via a penoscrotal approach. Subsequently, he underwent revision in August 2023 via an infrapubic approach with insertion of a Coloplast Titan Touch three-piece IPP. The system comprises two cylinders, a scrotal pump, and an abdominal reservoir [11]. The "Touch" model features a one-touch deflation mechanism for simplified use [11]. A pre-existing reservoir was drained and retained. He was catheterised post-operatively due to urinary retention. Although the initial trial without a catheter (TWOC) failed, a subsequent community TWOC was successful. Two weeks post-op, he was reviewed in the andrology clinic and advised to cycle the implant daily. There was no clinical evidence of post-operative infection.

**Figure 1:**
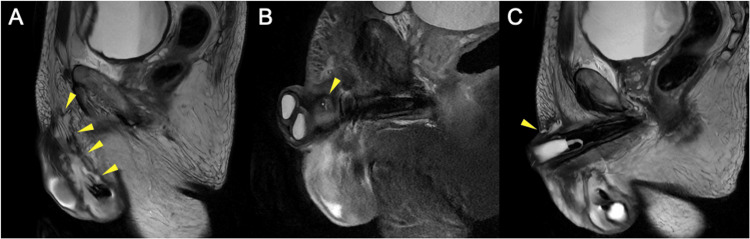
Sagittal MRI of the Penis Prior to Inflatable Penile Prosthesis Explantation (A) T2-weighted. High-signal fluid collection surrounding the prosthesis within the scrotum, extending superiorly towards the corpus cavernosum (arrowheads). (B) STIR. Small fistulous tract from the right corpus cavernosum (arrowhead), in continuity with the infected fluid collection (not shown). Induration of the scrotal and pubic soft tissues. (C) T2-weighted. Fistulous tract extending to the skin (arrowhead).

Almost two years later, in April 2025, the patient was admitted to a local hospital for upper gastrointestinal bleeding. During this admission, he developed purulent discharge from the glans penis, fluid leaking from the midshaft and pus discharging from the previous infrapubic wound site. Prior to the onset of infection, several unsuccessful and traumatic urethral catheterisation attempts had been made, with resistance encountered and suspected inadvertent partial balloon inflation within the urethra. This was followed by four days of external (conveen) catheter use, which may have exerted sustained local pressure and friction on the penile shaft. Collectively, these factors are suspected to have produced localised mechanical and ischaemic injury, predisposing to subsequent infection and fistula formation. MRI of the penis revealed oedema of the penis and perineum, fluid collections around the scrotal pump, and a 7 x 4 cm abscess around the glans (Figure 1). Examination following transfer of hospitals revealed a semi-inflated device, scrotal tenderness, right-sided penile abrasion, ulceration near the urethral meatus, and a sinus in the infrapubic region discharging pus.

**Figure 2:**
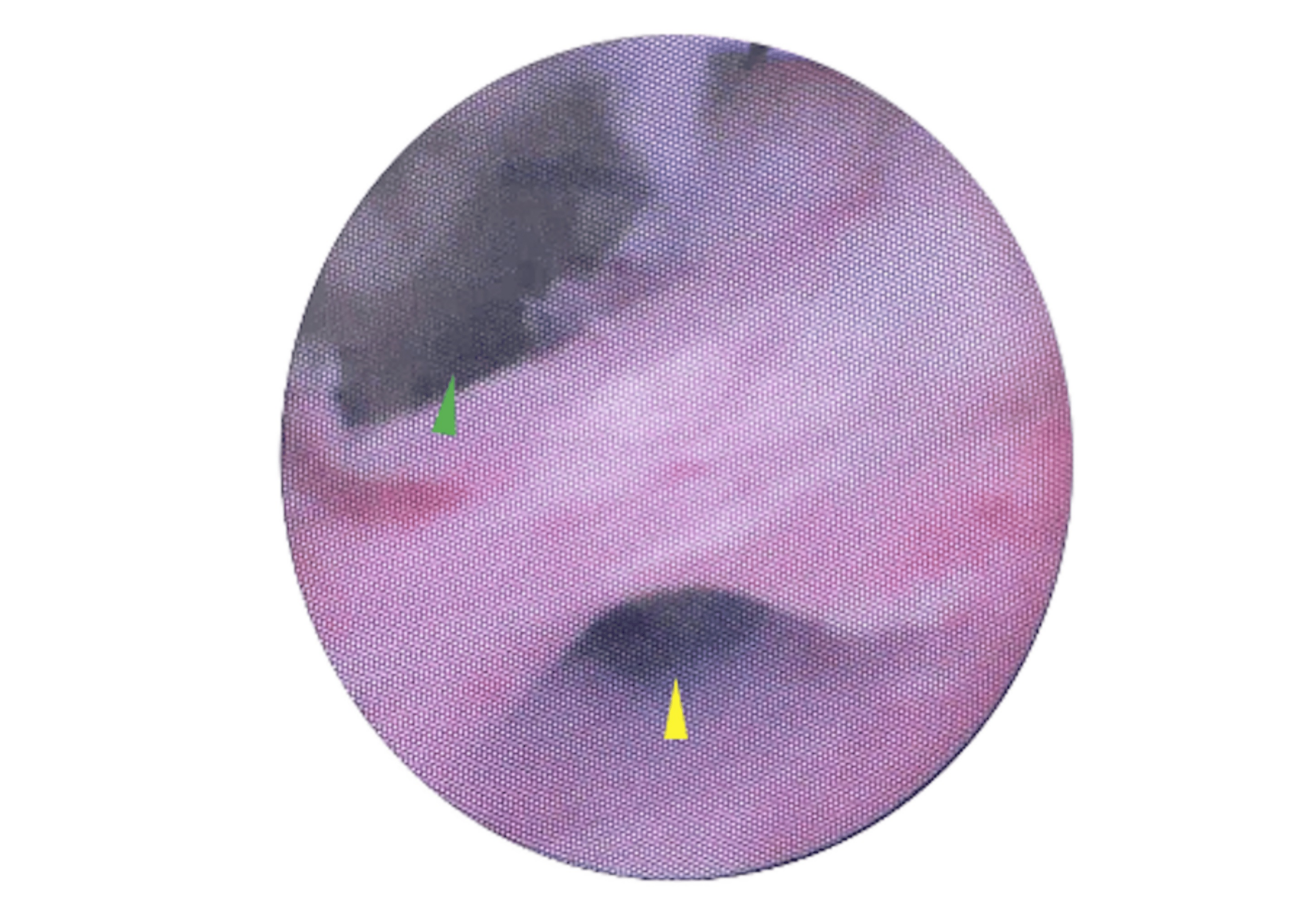
Intraoperative Flexible Cystoscopy View Demonstrating the Urethrocavernocutaneous Fistula The distal urethral lumen is visible at the lower aspect of the image (yellow arrowhead), while the larger defect superiorly (green arrowhead) represents the fistulous tract communicating with the corporal tissue and skin.

**Figure 3:**
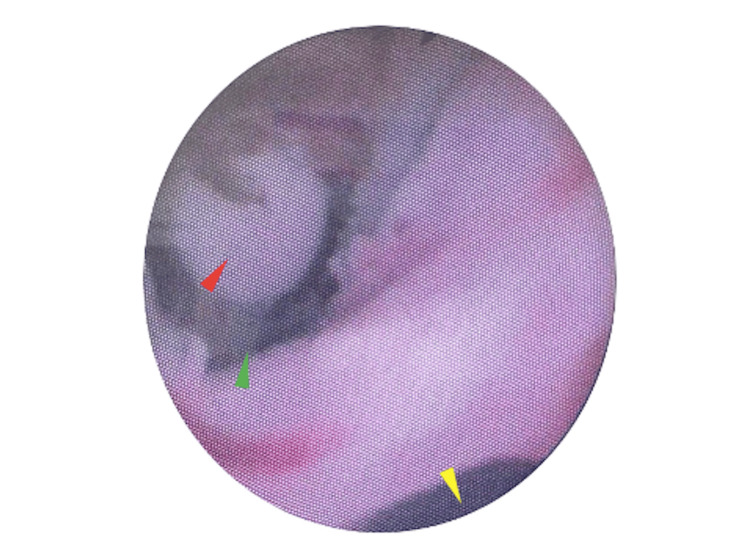
Intraoperative Flexible Cystoscopy View of the Urethrocavernocutaneous Fistula The distal urethral lumen (yellow arrowhead) is located at the lower aspect of the image. At the superior aspect, the fistulous opening is seen (green arrowhead) with the surgeon's gloved finger visible within the tract (red arrowhead), confirming the communication between the urethra, corporal tissue, and skin.

**Figure 4:**
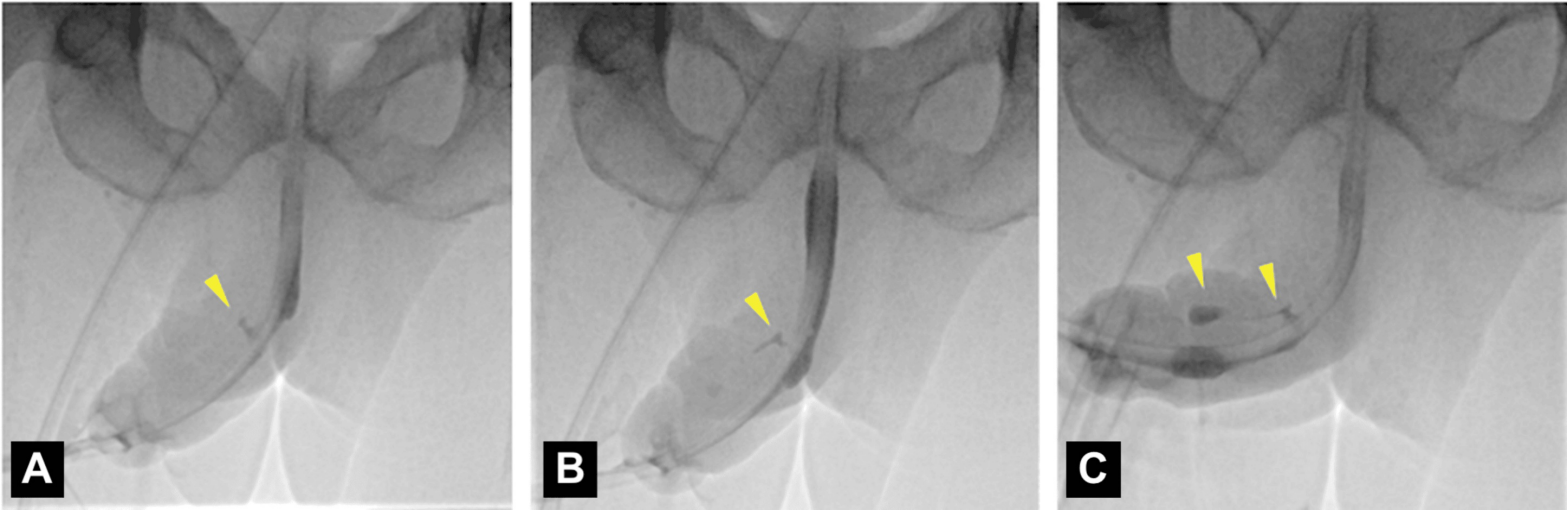
Urethrogram Eight Weeks Post-explantation (A–C), Performed Parallel to an Indwelling Urethral Catheter Contrast leakage from the mid-penile urethra into the right corpus cavernosum (arrowheads), confirming a fistula.

On 21 May 2025, the patient underwent explantation of the IPP. Intraoperative findings included a urethrocavernocutaneous fistula and purulent drainage. The prosthesis showed no mechanical failure, but on washout, fluid was observed exiting from both the mid-shaft fistula and urethral meatus. Flexible cystoscopy demonstrated a urethral defect 2 cm distal to the corporotomy (Figures 2, 3). It was felt to be unsafe to proceed with urethral repair via the peno-scrotal approach, given the unfavourable condition of the tissue, which was infected and friable. Given the patient was clinically unstable, suprapubic and urethral catheters were placed under cystoscopic guidance as a temporising measure. Cultures, taken intra-operatively, grew *Nakaseomyces glabratus*. The patient required HDU admission post-operatively. A urethrogram, performed three months post-operatively, confirmed persistent urethrocavernosal communication and contrast leakage into the soft tissues of the penis (Figure 4). The planned ongoing management is suprapubic catheter drainage; however, urethroplasty would have been preferred had the patient's fitness permitted.

## Discussion

Catheter-related trauma in patients with IPPs represents a significant but under-recognised source of morbidity. While infection and mechanical failure are established complications [3,12], both urethral and external catheters have been implicated in erosion, fistula formation, and ultimately device loss. To our knowledge, this is the first documented case of a urethrocavernocutaneous fistula complicating an IPP. The urethral opening of the fistula was most likely caused by traumatic urethral catheterisation, consistent with previous reports demonstrating that even a single episode of balloon inflation in the urethra can result in erosion and fistula formation [4,6]. In addition, the external wound corresponded anatomically to the site of external catheter placement, raising concern for pressure-related ischaemia, a mechanism supported by Arslan et al. and Jabbour et al. [5,9].

Several reports have emphasised the risks of urethral catheterisation in IPP patients. Vaidyanathan et al. described a diabetic spinal cord injury patient who developed urethral erosion and perineal fistula following repeated balloon misplacements [4], while Panuganti et al. highlighted that even short-term catheterisation in ICU patients with COVID-19 could lead to IPP erosion [7]. Hisasue et al. also reinforced the importance of cross-specialty awareness by reporting erosion in a patient with an indwelling catheter and an unrecognised prosthesis [6]. Historical data from Steidle and Mulcahy demonstrated urethral erosion rates as high as 56% in prosthesis patients requiring intermittent or indwelling catheters [13].

Although external catheters are often perceived as safer alternatives, recent cases challenge this assumption. Penile skin necrosis [5] and gangrene with a potentially fatal outcome [9,10] have been reported following their use. Our case highlights how internal and external mechanisms may act synergistically to produce severe morbidity.

Erosion of penile prostheses is a recognised complication, but subsequent fistula formation is rare. Botkin et al. reported a bulbar urethrocavernous fistula in a diabetic patient after IPP revision [8], and Siles et al. underscored both the rarity of such cases and the value of retrograde urethrography for diagnosis [14]. Brown et al. described tubing erosion into the urethra without prior catheterisation or infection, suggesting that multiple aetiologies may contribute [15].

In contrast to the aforementioned cases, our case involved sequential urethral and external catheter trauma acting through both mechanical and ischaemic mechanisms. This dual aetiology may explain the extent of soft-tissue necrosis and the complexity of the fistulous communication observed, features not emphasised in prior reports.

Management of infection, erosion, or fistula almost always requires explantation of the prosthesis, followed by irrigation and delayed reimplantation, if appropriate [16]. Although salvage procedures have occasionally been attempted in highly selected patients [17], they are rarely appropriate in the setting of friable or infected tissue. Our patient's intraoperative cultures also grew *Nakaseomyces glabratus*, which is notable as fungal infections in penile prostheses are uncommon but increasingly recognised in immunocompromised hosts [3].

This case underscores the importance of timely surgical intervention and interdisciplinary coordination. Despite MRI and urethrogram being performed, cystoscopy provided the most definitive diagnostic information. Ultimately, explantation with extensive washout and dual urinary diversion was required, consistent with expert opinion [16].

Existing guidelines from the American Urological Association (AUA) and the European Association of Urology (EAU) remain sparse regarding catheterisation risks in men with penile prostheses [18,19]. This lack of guidance is particularly problematic given that non-urologists are often responsible for catheter placement during acute admissions. As IPP implantation becomes increasingly common, clearer recommendations and greater awareness are needed to prevent avoidable complications.

In summary, catheter-associated trauma is a preventable yet underappreciated cause of IPP morbidity. Our case reinforces three key messages: both urethral and external catheters can cause significant harm, systemic co-morbidities compound local tissue injury, and a multidisciplinary, stepwise surgical approach with explantation and diversion of urine away from the urethra is the treatment of choice for infected prostheses with erosion or fistula formation.

## Conclusions

Catheter-associated trauma is an under-recognised but preventable cause of IPP complications. Both urethral and external catheters can precipitate serious outcomes, including erosion, necrosis, and fistula formation, particularly in patients with multiple co-morbidities and impaired tissue healing.

Management most often requires device explantation and urinary diversion, with reimplantation considered only once infection is resolved. Current international guidelines provide only general recommendations and do not address catheter use in IPP patients. Preventive strategies should include clear documentation of IPP presence in medical records, avoidance of unnecessary urethral catheterisation, and cautious use of external devices with regular inspection for pressure injury. Greater awareness and clearer guidance are needed to minimise avoidable harm in this high-risk population.
